# Potential effects of lowering the threshold of statistical significance in the field of chronic rhinosinusitis – A meta-research on published randomized controlled trials over last decade

**DOI:** 10.1016/j.bjorl.2021.11.004

**Published:** 2021-12-04

**Authors:** Pooja Thakur, Vivek Jha

**Affiliations:** a Maharishi Markandeshwar Medical College and Hospital, Department of Otorhinolaryngology and Head & Neck Surgery, Solan, India; b Maharishi Markandeshwar Medical College and Hospital, Department of Orthopaedics, Solan, India

**Keywords:** Randomized controlled trial, Clinical trials, Sinusitis, PubMed

## Abstract

**Objective:**

To evaluate the impact of change in *p*-value threshold from 0.05 to 0.005, on published Randomized Controlled Trials (RCTs) over the last ten years in the field of chronic rhinosinusitis.

**Methods:**

A search of the PubMed database from 1st January 2011 to 31st December 2020 was conducted to include all RCTs that used *p*-value to determine the effects of an intervention. Data extracted included *p*-values, type of intervention, publishing journal with indexing, registration, funding, and multi- or single center status. The proportion of primary endpoints having *p* < 0.005 was determined first, followed by those with *p* > 0.005 but <0.05. Logistic regression analysis was used to determine if any trial characteristic was associated with reporting of significant *p*-values.

**Results:**

In total, 168 primary endpoints were identified from 123 RCTs. On analysis, 80 had a *p*-value <0.05, i.e., statistically significant on conventional parameter. Out of these, 53.75% had a *p*-value <0.005, which would retain significance under the proposed threshold, and the remaining 46.25% to be reclassified as “suggestive”. None of the trial characteristics were contributing to reporting of conventional or proposed *p*-values on logistic regression analysis.

**Conclusion:**

Lowering the *p*-value threshold would render 46.25% of a decade of published RCTs results (in the field of CRS) to be reclassified as merely “suggestive” and not significant. Trial characteristics were not found contributing to reporting of *p*-value <0.005 or even <0.05.

**Level of evidence:**

II.

## Highlights

• *p*-value statistic has multiple demerits and limitations.
• Lowering the *p*-value threshold from 0.05 to 0.005 would heavily alter the interpretation of RCTs in the last ten years.
• Trial characteristics such as funding, single or multicentered status, or registration status, were not found contributing to reporting of a significant *p-*value.
• Scientific literature needs to do away with over-reliance on *p*-value and there is a requirement for alternate methods of interpretation of results.

## Introduction

Evidence-based medicine is the foundation of modern era health systems to help in gaining triple aim of improved care, health, and cost.^1^ It promotes the concept of applying the best available evidence from biomedical research, particularly obtained from the Randomized Controlled Trials (RCTs), into patient management.^2^ Being the gold standard of scientific research methodology, RCTs impose a major influence over clinical practice guidelines and management protocols. The basis of statistical analysis to determine superiority or non-inferiority of an intervention in RCTs lies with Null Hpothesis Statistical Testing (NHST) and *p*-value. Although RCTs are at the top of the evidence hierarchy, there has been a growing concern over the reproducibility crisis associated with them, owing to dependency on *p*-values.^3^ Limitations of the *p*-value must be understood as many fallacies have been associated with it, especially with its interpretation. Many authors have suggested that the non-reproducibility of results along with the high rate of false positives could be attributed to conventional *p*-value statistics.^4^, ^5^ *P* hacking, spurious false-positive results, data dredging, non-adequate power, publication bias, exaggerated effect sizes, “winner’s curse” etc. could be attributed to *p*-value statistics.^6^, ^7^, ^8^

Taking these demerits into consideration, the predominant application of *p*-value in its current defined form and relying completely on it for interpretation of results in RCTs can be misleading. In turn, it can drastically over- or under-emphasize the statistical significance of interventions tested and can affect patient care based on those results.^9^, ^10^ It has been stated that most often *p*-values are misinterpreted, overtrusted, and misused, and hence, there has been an increasing emphasis on modifications in the current *p*-value definition.^11^ Some have even suggested to completely abandon NHST and *p*-value thresholds.^12^ In fact, there has been a discussion on decisions of some scientific journals which are not promoting the use of statistical mathematics and *p*-values.^13^, ^14^, ^15^

In agreement with this, a large group of statisticians has proposed to adopt a new significance level by lowering the *p*-value from 0.05 to 0.005. Values ranging in between would be reclassified as “suggestive”. They have illustrated a reduction in the minimum false positive rate from approximately 33% to 5% on adopting this new threshold value.^16^ Recently conducted projects on replication of results in the field of psychology and experimental economics have also provided empirical evidence in support of success and better reproducibility with the proposed change in *p*-value threshold of 0.005.^17^, ^18^

Many researchers have tried to assess the impact of such alterations on existing evidence (RCTs) in the fields of general medicine, orthopedic trauma, and sports medicine.^19^, ^20^, ^21^ A major chunk of published RCTs in high-impact journals in these fields was shown to require reclassification from significant to “suggestive”. The impact of this new threshold *p*-value on Otorhinolaryngology has not been explored yet.

In Otorhinolaryngology, Chronic Rhinosinusitis (CRS) is one such prevalent condition that affects humans globally and constitutes a major public health issue.^22^ Hence, authors evaluated previously published RCTs on the subject of CRS and studied the impact of change in the *p*-value threshold from 0.05 to 0.005. The authors hypothesized that the proposed change in *p*-value threshold from 0.05 to 0.005 would cause a reduction in the proportion of statistically significant results in CRS randomized controlled trials.

## Methods

The authors searched PubMed from January 1, 2011, to December 31, 2020. The search string was as follows: (((CRS) OR (CRSwNP)) OR (CRSsNP)) OR (“chronic rhinosinusitis”).

Filters applied were Randomized controlled trial and English language. 2 authors (PT, VJ) independently assessed the articles for eligibility in two rounds. The screening process consisted of going through the abstract and title of the study. Following the first round of screening, studies eligible for full-text analysis were scrutinized.

**Figure 1:**
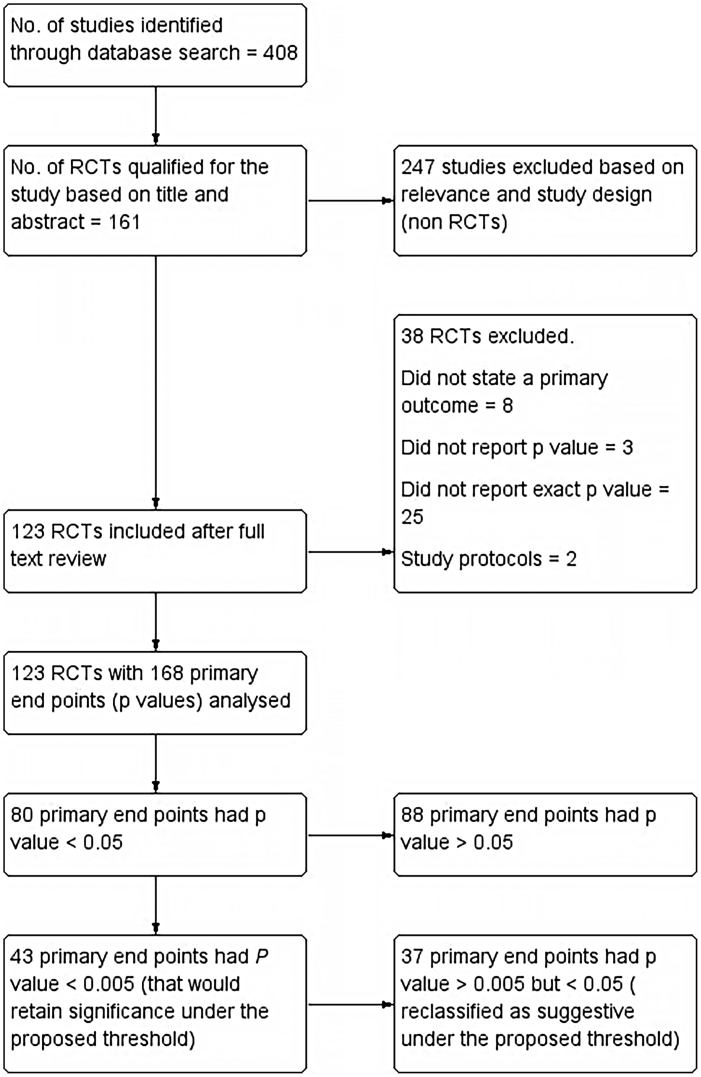
Flow diagram of study inclusion (RCT, Randomized Controlled Trial).

All Randomized Controlled Trials (RCTs) in PubMed database of last 10 years duration (1st January 2011–31st December 2020), that used a *p*-value to determine effects of intervention along with a clearly stated primary endpoint, were included. If a study had more than one primary endpoint, all *p*-values for multiple primary endpoints were also included. Single-group trials, pooled analyses, RCTs without *p*-values, cross-over studies, study protocols, and RCTs that used Bayesian or non-inferiority analyses were excluded. Summary of included studies as well as those excluded is reported as per PRISMA (Preferred Reporting Items for Systematic Reviews and Meta-analyses) guidelines and has been shown in the flow chart (Fig. 1).

Following details were extracted from each trial. The number of primary endpoints and their respective *p*-values, type of intervention, name of publishing journal with its indexing, registration status, source of funding, whether the trial was a multicenter or single-center, and year of publication. Primary endpoints without an exact *p-*value were excluded. The type of intervention was categorized as drug, surgical procedure, or others. Indexing status was segregated as MEDLINE or PubMed. Studies providing a trial registration number were categorized as registered and those without were considered not registered. Data were extracted in a blinded fashion and in duplicate and any disagreements among investigators were settled with consensus.

The primary endpoints were segregated into three categories. First of all, the proportion of endpoints with a *p*-value less than 0.05 was estimated. These were classified as “significant conventionally”. Next, the proportion of primary endpoints with *p* < 0.005 was estimated and they were classified as “still retaining statistical significance”. The proportion of primary endpoints with *p* > 0.005 but <0.05 was classified as “suggestive”. Additionally, the proportion of primary endpoints with *p* < 0.01 was also determined. Next, we attempted to find out if any of the trial characteristics including registration status, funding, multicenter/single-center, and type of intervention studied was associated with reporting at least 1 primary endpoint with a *p*-value less than 0.005 while adjusting for other trial characteristics. A binomial logistic regression model was used for this purpose.

### Statistical analysis

IBM SPSS software v.26 was used for statistical analysis. The primary results of the study including the proportion of endpoints retaining statistical significance under the new threshold *p*-value were expressed in percentages and frequencies. Trial characteristics of included RCTs were also expressed using descriptive statistics. To find out if any trial characteristic was predictive of reporting at least 1 primary endpoint with a *p*-value less than 0.005, a binomial logistic regression model was used. The binomial logistic regression model included all trial characteristics (registration status, funding, multicenter/single-center, and type of intervention studied) as independent predictor variables. The dependent variable was whether the trial retained significance under the new threshold (*p* < 0.005). We also ran another logistic regression model to assess if trial characteristics were associated with significant results under the conventional *p*-value threshold (*p* < 0.05). The same set of trial characteristics were used as independent predictor variables.

## Results

The PubMed search retrieved 408 articles, out of which 161 articles qualified for the present study based on Title and Abstract. 161 articles were further evaluated for inclusion as well as exclusion criteria and 13 articles were excluded. Those excluded were mostly which did not state a primary outcome (8). Three did not report *p*-values and two were study protocols. On further analysis, another 25 articles were excluded as they failed to report the exact *p*-value. 168 primary endpoints were identified from the final included 123 articles.

Primary results: Out of 168 primary endpoints, 80 had a *p*-value <0.05, being statistically significant conventionally. Out of these, 43 (53.75%) had a *p*-value <0.005, which would retain significance under the proposed threshold. Only 30 of 123 included trials had all primary endpoints that met the new threshold of *p* < 0.005. Of conventionally significant endpoints, 37 (46.25%) would be reclassified as suggestive under the proposed threshold. We also evaluated threshold of *p* < 0.01 and found that 47 (58.75%) primary endpoints reported *p* < 0.01.

Trial characteristics: Patient-reported outcome measure/scores was the commonest reported primary endpoint (112/168; 66.67%). Drugs were the major type of intervention (71/123; 57.72%), followed by surgical procedures (28/123; 22.76%). Out of total trials included the majority (104; 84.55%) were single center studies. The funding source was mentioned in 51 trials (41.46%) with 11 of them being multicenter trials. Only 37 trials (30.08%) had themselves registered to provide registration numbers. The majority of trials were published in high-impact factor journals, the commonest being International Forum of Allergy and Rhinology (27), American Journal of Rhinology and Allergy (19), Rhinology (9), and American Journal of Otolaryngology (6). Interestingly, all included trials were published in journals indexed in MEDLINE.

Logistic regression analysis was conducted to identify if trial characteristics were predictive factor for at least one primary endpoint retaining statistical significance under the new threshold. Trial characteristics included in the model were registration status, funding status, type of intervention, and whether the study was conducted at single or multiple centers. As evident from Table 1, none of the trial characteristics were found to be significantly contributing to the prediction of at least one primary endpoint retaining statistical significance. A point to note, the *p*-value for the test of significance in the model was kept <0.005, and none of the *p*-values were found significant. Furthermore, *p*-values in the model were all found to be more than 0.05. In other words, even if we were to test significance at the conventional value of *p* < 0.05, the results would all still be the same. In addition, we ran logistic regression again to assess if trial characteristics were associated with trials reporting at least one primary endpoint as significant (as per conventional threshold *p* < 0.05). As evident in Table 1, none of the trial characteristics were found to be a significant predictor.

**Table 1:** Logistic regression analysis of trial characteristics and reporting a significant *p*-value.

	*p* < 0.005			*p* < 0.05		
	Significance	Odds ratio	95% CI odds ratio (lower, upper)	Significance	Odds ratio	95% CI odds ratio (lower, upper)
Registration (un-registered)	0.190	0.536	0.211, 1.362	0.541	1.326	0.537, 3.274
Funding (non-funded)	0.380	0.676	0.282, 1.621	0.156	0.548	0.239, 1.259
Multicenter	0.842	1.116	0.379, 3.287	0.720	1.206	0.433, 3.359
Intervention (drugs)	0.984	1.010	0.355, 2.880	0.990	1.006	0.390, 2.599
Intervention (procedures)	0.720	1.254	0.364, 4.325	0.750	1.202	0.387, 3.737
Intervention (others)	0.901	–	–	0.922	–	–
Constant	0.666	0.782	–	0.668	1.259	–

CI, confidence interval.

## Discussion

The primary objective of this study was to evaluate the impact of change in *p*-value threshold from 0.05 to 0.005, on published RCTs related to chronic rhinosinusitis. RCTs were selected as the unit of the current study as they are the source of the highest-level evidence in biomedical literature influencing clinical guidelines. The purpose of taking only primary endpoints into account was based on the fact that RCTs are powered on these endpoints. In the present study, the authors found that only 53.75% of primary endpoints of trials studied, which were statistically significant based on conventional *p*-values, would still retain significance on lowering the value to *p* < 0.005. However, 46.25% of primary endpoints, that is almost half, would be reclassified as “suggestive”. This shift of results from significant to “suggestive” would have a major impact on the interpretation of these trials and would warrant caution on acceptance of their results, as the probability of chance affecting these results increases.

The concept of null hypothesis significance testing was introduced to distinguish the relevant and interesting observations from the background noise.^23^, ^24^ Conventionally, an arbitrary value of 0.05 (*p*-value) is taken as a cut-off, when applying NHST, to classify the observations into statistically significant and not significant.

Multiple demerits have been associated with the rampant and predominant use of this *p*-value. These include *p* hacking, spurious false-positive results, underpowered trials, and non-reproducibility of results.^4^, ^25^, ^26^ Lack of reproducibility has led to a concern over new claims based on *p*-value significance. There is a little incentive to promote replication research and even much less inclination towards negative results (on replication) to be highlighted.^27^ This non-reproducibility results in a gap between statistical results and scientific conclusions, thus leading to wastage of funding and questioning the credibility of studies. The *p*-value is often misinterpreted by equating it with the strength of an association. However, very low *p*-values can be seen in situations where one has a tiny effect size but a large sample size. Similarly, a small *p*-value does not necessarily imply a major clinical or biological relevance.^28^ This is especially true in cases of underpowered studies as the *p*-value exhibits low reliability and wide sample to sample variability unless statistical power is high.^8^ *p* hacking, another downside of NHST is a type of researcher-driven publication bias, known as the inflation bias, and it implies selective reporting of only those statistical analyses which produce significant results.^29^, ^30^ *p* hacking is associated with false-positive results as well as HARking i.e., generation of hypothesis after results are known.^31^ It has also been emphasized that *p*-value studies (NHST) are often applied as a “mindless null ritual”^32^ and tend to override the importance of the power of the study and mislead owing to the neglect of pre-data probabilities.^33^ A proposal to lower this *p*-value threshold from 0.05 to 0.005 is aimed to reduce various shortcomings associated with *p*-value statistics as well as increase the validity of results. Varied results on applying this new value have been observed in different fields of biomedical research. If the *p*-value threshold is lowered to 0.005, a significant shift of almost one-third of the results of existing biomedical research from statistically significant to “suggestive” has been reported by Chavalarias D et al.^9^ Evaluation of proposed change in *p*-values in orthopedic sports medicine suggested that only 38.9 primary endpoints would retain significance whereas there was a reclassification of 61.1% endpoints into “suggestive”.^21^ In another analysis involving orthopedic trauma randomized controlled trials, it was found that only 20.2% of endpoints would retain statistical significance as per the proposed *p-*value threshold.^20^ However, among the clinical trials conducted in the field of general medicine, a better proportion of primary endpoints i.e., 70.7%, was found to retain statistical significance under the proposed *p*-value threshold.^19^ Our results lie somewhere in between i.e., 53.75% endpoints would retain statistical significance.

This wide variability in results could be attributed to multiple factors including sample size, power, and methodology. An important difference between the study done by Wayant et al.^19^ and others including the present study is the proportion of multicenter trials. The trials analyzed by Wayant et al.^19^ included 89.2% multicenter trials whereas others had 3%,^21^ 27.1%,^20^ and 15.45% (present study) multicenter trials. Multicenter trials score better over the single-center in terms of sample size, generalizability, and reliability.^26^

A significant observation to note in the present study is that only 30.08% (37/123) trials were prospectively registered, providing valid registration information. The other study in orthopedic sports medicine reported an even lower proportion (11.1%) of registered trials.^21^ It has been suggested that studies lacking pre-registered protocols may end up with non-systemic and non-transparent analysis plans.^11^ Such biomedical research, including the randomized trials, with a lack of systematic exploration, may result in selective reporting and biased conclusions. Hence, it has been proposed that pre-registration of all hypothesis testing studies based on null hypothesis significance testing should be done to ensure systematic analysis.^33^

There has been supporting evidence to highlight that there is a disproportionate publication of statistically significant results in the journals with high-impact factors.^34^, ^35^, ^36^, ^37^, ^38^ This has led to what is known as the “File drawer effect”.^39^ Although the other authors had analyzed only the high-impact journals in their respective fields,^19^, ^20^, ^21^ we searched the whole of the PUBMED database to characterize the trials based on publishing journals and their indexing status, and found that the majority of trials were published in various prestigious high-impact journals, all indexed in MEDLINE.

Funding forms yet another important characteristic related to RCTs and a researcher’s performance is assessed by a funding agency based on their publications in the high-impact factor journals.^40^ Directly or indirectly, this has an influence on the researcher to selectively highlight and publish statistically significant findings. In fact, Wayant et al.^19^ in their analysis found a funding source (both industry and non-industry) for almost 99% of trials, and those with industry funding were more likely to report the primary endpoints which would still retain statistical significance (*p* < 0.005) (OR = 7.87, 95% CI 3.14–19.71). The authors in the current study did not find funding to be a predictive factor for reporting *p*-value <0.005 in trials. In fact, none of the trial characteristics assessed were found to be contributing to reporting a *p*-value less than 0.005. Furthermore, the fact our results also show that trial characteristics including funding were not associated with reporting significant results based on the conventional threshold (*p* < 0.05), highlights the quality and integrity of research in the field of CRS, which is commendable. Evans et al.^21^ in their study found 43.2% trials with a funding source. Similar to the current study, they also conducted logistic regression analysis and found that none of the trial characteristics including funding source and type of intervention were contributory to reporting *p*-value <0.005.

Although lowering the *p*-value threshold to 0.005 cannot correct the other deep-rooted issues associated with NHST, it can act as a simple, convenient, and compliant modification with the existing training of researchers. One should also be aware of the collateral damage associated with the new *p*-value, like exaggerated effect sizes, escalation of publication biases, and decreased clinical relevance of selective endpoints.^11^ Additionally, lowering the *p*-value threshold would require much larger sample sizes and consequently added trial costs and study time. The authors were curious to explore the impact of lowering the *p*-value threshold to 0.01 in our study and found that 58.75% of primary endpoints still retained their significance. Although, a significant percentage of research findings would be under scrutiny, based on the new threshold *p*-value (<0.005), it is interesting to note that there is not much difference in the fraction of studies retaining significance with the proposed threshold of *p* < 0.01 and *p* < 0.005. However, the *p*-value of 0.01 would definitely require a smaller sample size.

In the authors’ opinion, the *p*-value fallacy is just the tip of an iceberg, multiple other issues of data dredging, underpowered studies, arbitrary variable dichotomization, to name a few, need to be dealt with. Advance statistical analysis training and usage wherever appropriate would lead the researchers to better scientific conclusions.

### Strengths and limitations

While other studies extracted data from a few (3) high-impact journals, the current study scouted the complete database including multiple high-impact journals ensuring better generalizability. In addition, a literature search spanning a decade of published literature is another strength of this study, while other studies were restricted to a duration of one-two years. However, limiting the research to a single database is a major limitation of this study.

## Conclusion

Lowering the *p*-value threshold would render 46.25% of a decade of published RCTs results (in the field of CRS) to be reclassified as merely “suggestive” and not “significant”. Trial characteristics were not found contributing to reporting of *p*-value <0.005 or even <0.05.

## Funding

This research did not receive any specific grant from funding agencies in the public, commercial, or not-for-profit sectors.

## Ethics committee approval

The unit of the study consisted of published randomised controlled trials in the relevant field. The study does not involve human or animal participants. There was no search of any patient related records or documents for the purpose of the study. Hence, ethics approval is not required for the present study.

Since the study does not involve any human participant or any patient specific identification/details/information, there was no need for the patient consent form.

## Authorship contributions

Concept: Pooja Thakur, Vivek Jha; Design: Vivek Jha; Data collection: Pooja Thakur; Data analysis and Interpretation: Pooja Thakur, Vivek Jha; Literature search: Pooja Thakur; Writing: Pooja Thakur.

## Conflict of interests

The manuscript submitted does not contain information about medical device(s)/drug(s).

No copy righted material has been used in the manuscript.
